# Supplementary material for: Interaction between Mas1 and AT1RA contributes to enhancement of skeletal muscle angiogenesis by angiotensin-(1-7) in Dahl salt-sensitive rats
Source: PLoS One. 2020 Apr 23;15(4):e0232067. doi: 10.1371/journal.pone.0232067 (PMC7179868; doi:10.1371/journal.pone.0232067)

Fig S1

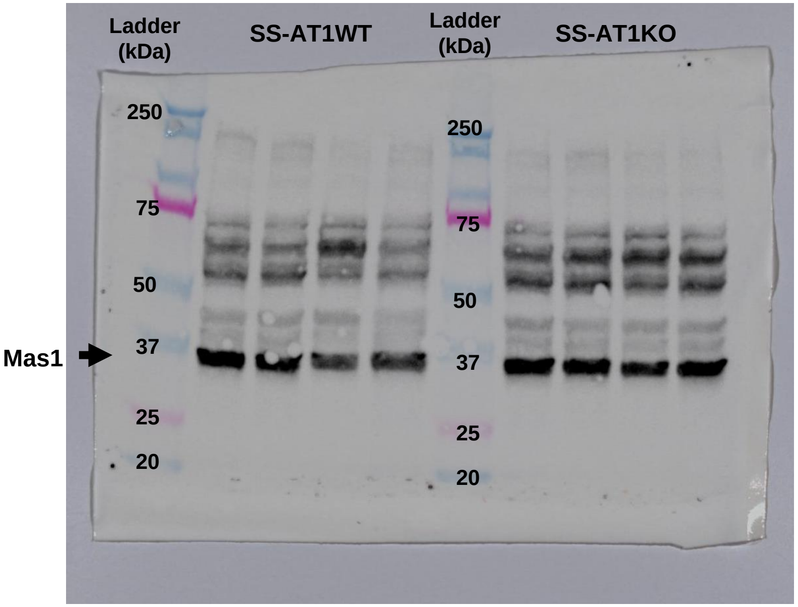
Chemiluminescence with colorimetric marker
(with labels)
Ladder
(kDa)
SS-AT1WT
Ladder
(kDa)
SS-AT1KO
250
75
50
37
25
20
250
75
50
37
25
20
Mas1

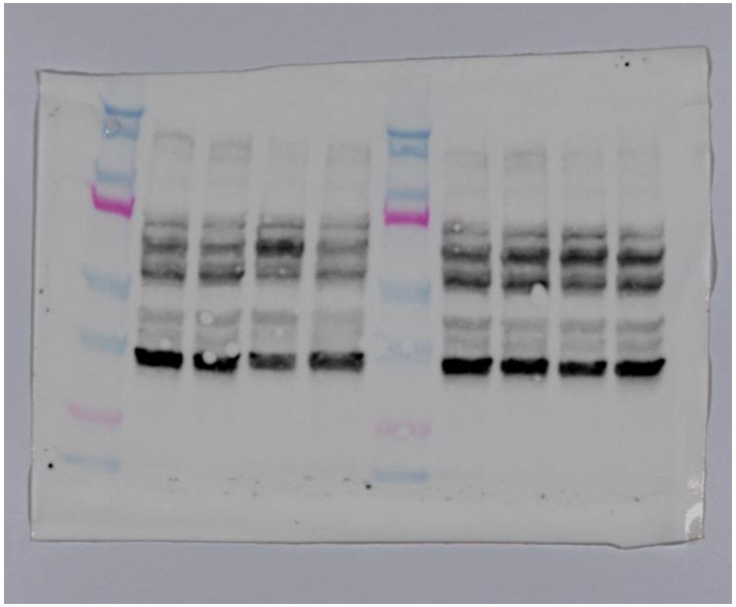
Chemiluminescence with colorimetric marker
(no labels)

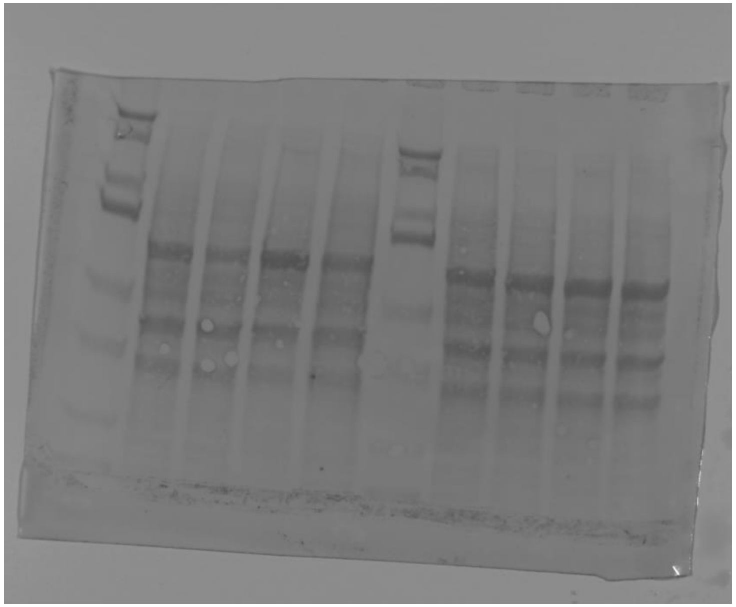
Ponceau S colorimetric protein stain

Chemiluminescence with colorimetric marker (with labels)

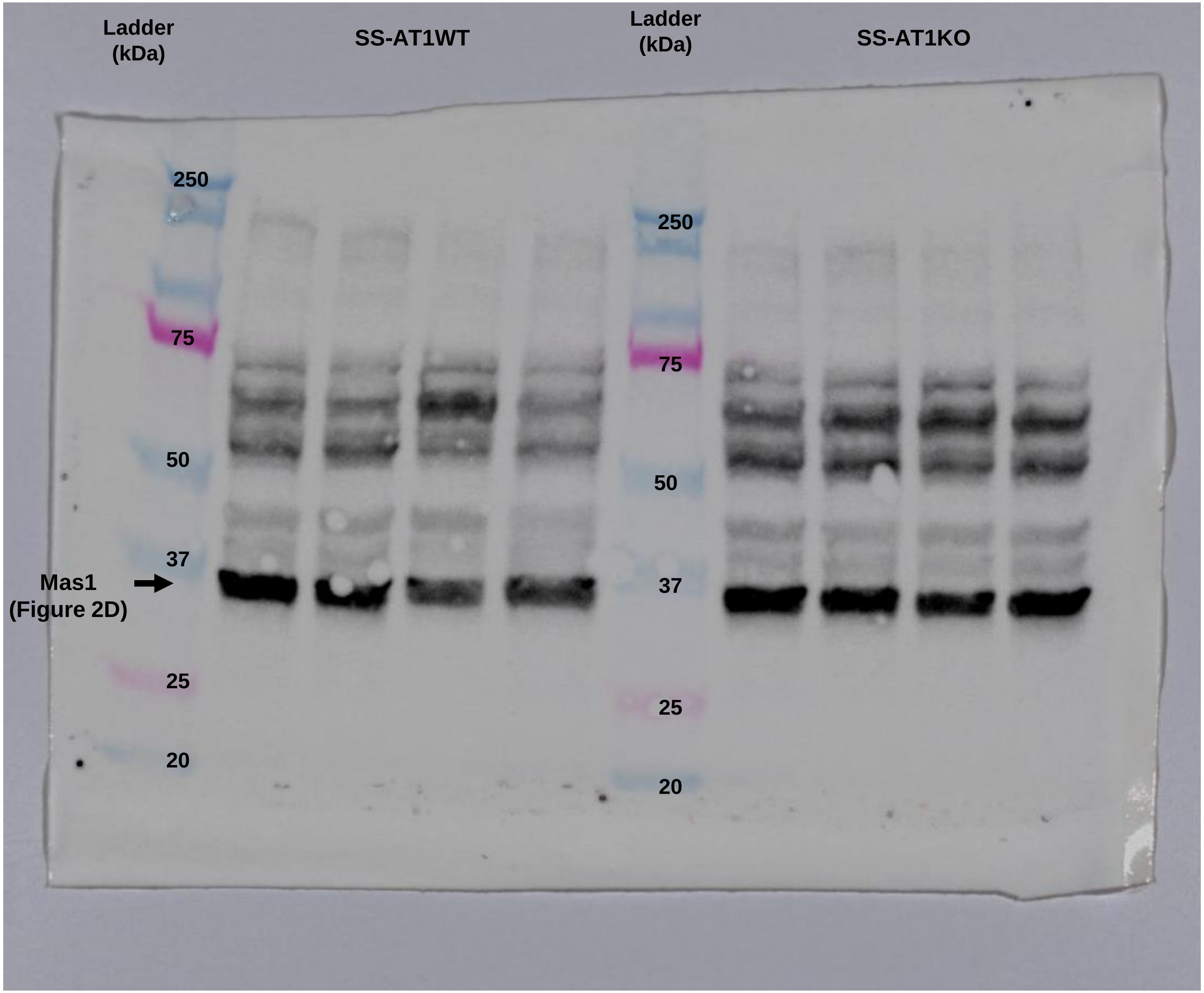

Chemiluminescence with colorimetric marker
(no labels)

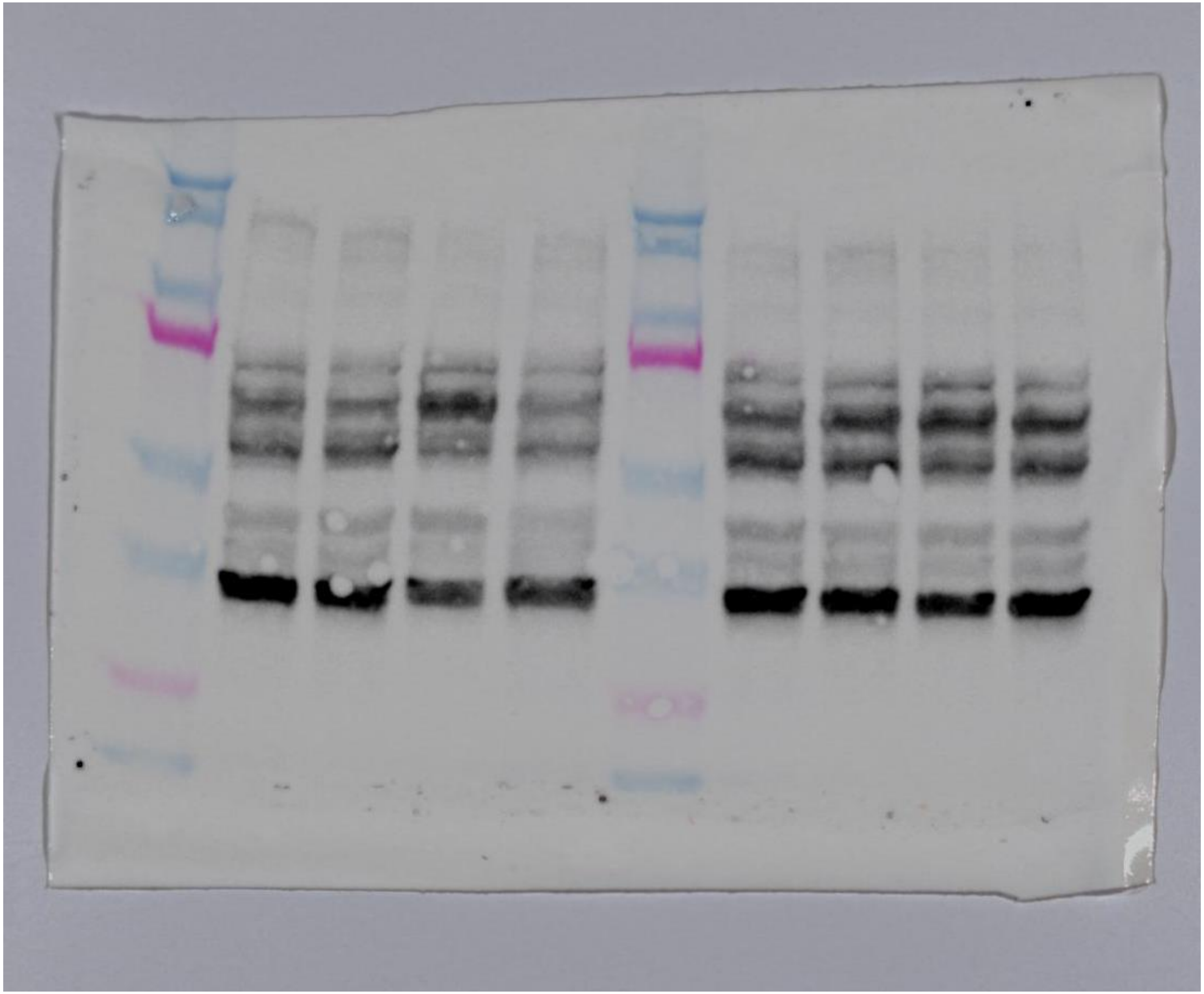

Ponceau S colorimetric protein stain

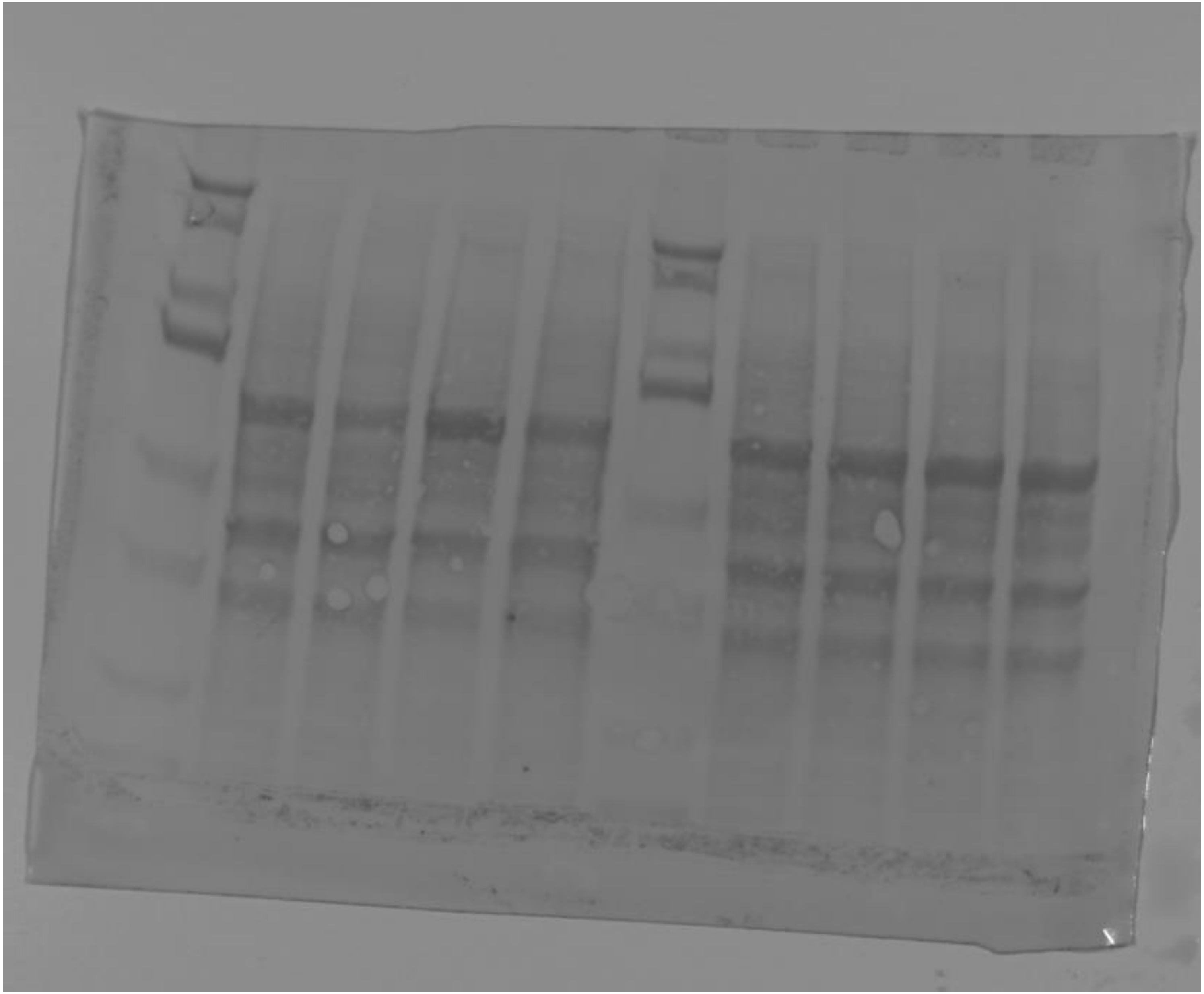

Supplement: S1 Raw Images — (PDF) [file pone.0232067.s001.pdf]
