# Supplementary material for: Interaction between Mas1 and AT1RA contributes to enhancement of skeletal muscle angiogenesis by angiotensin-(1-7) in Dahl salt-sensitive rats
Source: PLoS One. 2020 Apr 23;15(4):e0232067. doi: 10.1371/journal.pone.0232067 (PMC7179868; doi:10.1371/journal.pone.0232067)
Supplement: Attachment — Submitted filename: Response to Reviewers 27.3.20.docx [file pone.0232067.s007.docx]

**Reviewer #1:**

1.) Fig. 2C. There appears to be no mRNA of Mas1 present in EC’s. What we’re the number of PCR cycles that was needed to detect a signal? And was there any Mas1 protein? This needs to be answered. It should be possible to show protein levels since the authors used antibodies for immuno-precipitation of Mas1. Please also provide adrenal gland gene expression from both WT and KO.

**Thank you for the opportunity to better communicate this data. We have replotted each transcript in Figure 2C on its own axis to make it clearer that Mas1 mRNA was present in both SS-AT1WT and SS-AT1KO cells. We have included the CT values in Supporting Information (S2 Table), with subsequent supplemental material renumbered correspondingly. We have also added a western blot for Mas1 protein as Figure 2D-E. Uncropped images are available in Supporting Information (S1 raw images). We have added a corresponding Immunoblotting section to Methods. The following was added to the text:**

**Methods section, page 8-9, line 201-213:**

**Immunoblotting:** SS-AT_1_WT and SS-AT_1_KO rat cardiac microvascular endothelial cells (RMVECs) (Cell Biologics) grown according to company protocol were brought to 70-90% confluency. Media was aspirated and cells were scraped in MPER buffer (Pierce 78501) containing Protease Inhibitor (Roche 11697498001. Cells were lysed with a 21-gauge needle and assayed for protein concentration with MicroBCA kit (Pierce 23235). Thirty-five micrograms of protein from each sample was loaded on a 10% TGX PAGE gel (Biorad) and transferred to nitrocellulose. Blot was blocked overnight in 5% NFDM (Biorad 1706404) and 1% BSA (Sigma A7906). Blot was incubated with Mas1 primary antibody (Santa Cruz sc-135063) at 1:1000 dilution overnight at 4 degrees C. Blot was rinsed and incubated with goat anti-rabbit HRP-conjugated secondary antibody (Biorad 1706515) at 1:5000 dilution for 1 hour. Blot was visualized with SuperSignal West Pico Chemiluminescence Substrate (Pierce 34080). Membrane was imaged on ImageQuant LAS 500 (GE Healthcare Life Sciences) and images were analyzed using ImageJ software (<https://imagej.nih.gov/ij/>).

**Results section, page 16, lines 404-407:**

Western blot confirmed the presence of Mas1 protein in both SS-AT1KO and SS-AT1WT endothelial cells (Fig 2D; complete blot found in S1 raw images). No differences were detected in Mas1 protein expression between the two groups (Fig 2E; p = 0.44 via 2-tailed t-test).

**Figure 2 Caption, page 16, line 392-396:**

D. Western blot for Mas1. Mas1 was present in both SS-AT1WT and SS-AT1KO endothelial cells; each lane contains protein from a separately cultured plate of endothelial cells. E. Relative quantification of Mas1 in SS-AT1WT and SS-AT1KO EC lysates. No difference was detected between these groups (n = 4; p = 0.442 via Students t-test).

2.) It is a bit strange to observe that WT show no angiogenic response to electrical stimulation. It makes one wonder what we are looking at. What happens in WT rats (no SS)? Does electrical stimulation elicit angiogenesis in such a model, and is this promoted by Ang-(1-7) and AngII? Or do we per se need a low renin model? If so, why is that? Are sufficient amounts of free AT1R needed perhaps, if one wishes to observe any Ang-(1-7) effect (through interaction of Mas with AT1R, or even binding of Ang-(1-7) to AT1R)? In SS rats Ang II levels are indeed low. It is important to test this.

**This is a good question, and we appreciate the opportunity to better communicate why we chose this model. We have added the following to the introduction, page 4-5, lines 89-104:**

We have established an animal model of skeletal muscle angiogenesis in which one hind limb is electrically stimulated to produce rhythmic muscular contractions over the course of seven days, resulting in increased microvessel density in angiogenically competent rats [1]. In this model, we have observed that modulation of the renin-angiotensin system affects angiogenesis. In particular, suppression of the renin-angiotensin system genetically[2,3], pharmacologically[3-5], or via environmental factors[2] impairs angiogenesis. We recently observed that low doses of angiotensin II and angiotensin-(1-7) have convergent, proangiogenic effects in this model of impaired angiogenesis [4].

In this study, we investigate the restoration of normal microvascular function in the Dahl salt-sensitive rat, a genetic model of low renin-angiotensin system activity and impaired angiogenesis, to test the hypothesis that proangiogenic Ang-(1-7) signaling is dependent on the presence of AT1R. Using wild type Dahl salt-sensitive (SS-AT1WT) rats and Dahl salt-sensitive rats with a novel mutation in AT1R resulting in an early truncation and loss of function (SS-AT1KO), we assess the consistency of Ang-(1-7) induced effects at the physiological level using a hind-limb model of angiogenesis, at the cellular level via endothelial cell tube formation, and at the molecular level via qPCR and tandem mass spectrometry-based proteomics.

3.) It is stated that the inability of losartan to affect Ang-(1-7) effects suggests that the ligand does not need to bind AT1R. This sounds logical when assuming that AT1R is in the same conformation as it would be when responsive to AngII. However, what if Mas1-associated AT1R has a different conformation, allowing activation by Ang-(1-7) (and antagonism by A779), and lowers losartan binding affinity? In fact, Mas was thought to be the AngII receptor when characterized as such for the first time in 1988 (Jackson et al. Nature 335). Probably Mas levels regulate AT1R levels and affinity? See also Gaidarov et al. Cell Signal. 2018, who present data that dismiss Mas as a binding partner for Ang-(1-7). This possibility should be taken into account. It can explain some of the older findings in which Ang-91-7) was identified as an AT1R antagonist (Mahon 1994, Gironacci 1999). In addition, the AT1R – Mas interaction appears to be important, and Ang-(1-7) is a tool to explore it. However, does this imply a physiological role for Ang-(1-7)? It is important to address these questions in the present manuscript. The comparison of Ang-(1-7) effect in Dahl SS vs. a WT rat strain might indicate if AT1R that is already occupied by AngII is in any way susceptible to modulation. If this is not the case it might be that Ang-(1-7) is indeed replacing AngII in SS rats to activate AT1R. In summary, it is still not clear if Mas is the direct binding partner of Ang-(1-7) (previous binding studies are not very convincing), but certainly the interaction of Mas with AT1R appears to be very important. The present and previous study of the authors indicate that AngII and Ang-(1-7) signaling pathways converge, providing evidence that the heptapeptide is in fact interacting with AT1R itself. Perhaps as a partial agonist, depending on the conformation of the receptor, which is regulated by Mas?

**This is a thoughtful and reasonable set of concerns and comments. We have added the following to the discussion section, page 26-27, lines 608-629:**

An important limitation of this study is the assumption that Ang-(1-7) does not exert its proangiogenic effects via binding to AT1R. This assumption is based upon our previous finding that the proangiogenic effects of Ang-(1-7) are blocked by the Mas1 antagonist A779 but not by the AT1R antagonist losartan [4]. It is possible that yet to be understood complexity will invalidate this assumption. For instance, an altered conformation of heterodimerized AT1R could allow Ang-(1-7) to bind AT1R despite the presence of losartan. However, note that the convergent, proangiogenic effects of AngII in our model were attenuated in the presence of losartan [4]. Unless AngII and Ang-(1-7) act via separate sets of AT1R, binding of AT1R by Ang-(1-7) in the presence of losartan is an unlikely mechanism for these losartan-independent proangiogenic effects. Given that the strongest evidence for Ang-(1-7) binding AT1R is displacement of AngII [6,7], we do not believe that binding of Ang-(1-7) to a separate set of AT1R that are unaffected by losartan is responsible for the proangiogenic effects observed in our model. Note that we do not discount AT1R binding as a mechanism for other effects mediated by Ang-(1-7). In fact, this possibility is of ongoing interest in the field. For instance, there is evidence that Ang-(1-7) can act as a biased ligand in cells expressing AT1R but not Mas1 [8]. There is also recent work suggesting that Ang-(1-7) does not interact directly with Mas1 [9]. This is in conflict with work demonstrating that Ang-(1-7) exerts effects via Mas1 [10], including in cells expressing Mas1 but not AT1R [11] and work that demonstrates Ang-(1-7) induced Mas1 internalization [12]. Given the ever-growing complexity of the renin-angiotensin system [13,14], including transactivation of Mas1 downstream of AngII [15], it seems unlikely that the effects of Ang-(1-7) are mediated via a single receptor. It will be important to reassess this work and other work in the field as our understanding of this complexity grows.

4.) Table 1: As it appears no Bonferroni correction was used for the number of genes that was included. Should that not be done?

**The hypothesis we set out to test with the qPCR array data was whether the overall changes in expression were different between groups, which we tested using a Deming regression (Fig. 5). Because we are also reporting data about individual transcripts, it is appropriate to apply a correction for multiple comparisons. We have applied a modified Hochberg step-up procedure and modified both the text and Table 1 to reflect this.**

**Results, Page 20, Lines 478-481:**

After correction for multiple comparisons via modified Hochberg step-up procedure [16] with FDR set at 0.05, significant changes were detected in the expression of 5 genes in SS-AT1WT cells but only 1 transcript in SS-AT1KO cells.

5.) For studying the protein complex MAS1 was co-precipitated with proteins that were bound upon stimulation with Ang-(1-7). A lot of proteins appear to bind MAS1, which is puzzling. Is this in agreement with the structure of MAS1? Or do the various proteins complex which each other. In order to support these findings it seems necessary to explore in the available protein sequence libraries if certain sequences can explain the results. It might also help to distinguish the relevant findings from those that are merely supported by statistical analysis.

**Cryolysis stabilizes large protein complexes, facilitating their isolation together and enabling minimal loss in the complex.  This was completed as in our previous manuscript [****4****].  In that manuscript it was also found that cross-linker could not be used because it blocked the MAS1 antibody; therefore, we utilized the cryolysis method based upon the Thermo Scientific Co-Immunoprecipitation Kit Cryolysis protocol (Thermo Fisher 14321D).  Co-immunoprecipitations using this cryolysis provide a deeper magnitude of identification into the protein binding complex than simple co-immunoprecipitation.  This does not mean that all of the proteins in the complex bind directly to the receptor but rather that they are part of the complex of interacting partners.  We have clarified this in the text as below.**

**Results, page 21, lines 493-499:**

The technique used here is cryolysis followed by co-immunoprecipitation as described in our previous study [4] and in the reagent manufacturer’s user guide (Thermo Fisher 14321D). Cryolysis stabilizes large protein complexes, facilitating their isolation together and promoting minimal loss in the complex. This provides a deeper magnitude of identification into the protein binding complex compared to simple co-immunoprecipitation. Note that this means identified proteins do not all bind directly to the target, in this case Mas1, but rather that they are part of a complex of interacting proteins.

Minor concerns:
Legends are found in the middle of the body text. I guess they should be following at the end?

**In the submission guidelines available on the PLOS ONE website, the following instructions are found: *“Figure captions are inserted immediately after the first paragraph in which the figure is cited. Figure files are uploaded separately.”*** [**https://journals.plos.org/plosone/s/submission-guidelines#loc-style-and-format**](https://journals.plos.org/plosone/s/submission-guidelines#loc-style-and-format)**. Most recent access on 3/21/2020. We believe the current arrangement of our manuscript adheres to this instruction.**

**Reviewer #2:**

1. It is necessary to discuss about the proteins binding to Mas in response to A1-7 in AT1KO ECs. That could explain how the AT1-independent pathway of A1-7-Mas stimulation would work.

**Please see response to 2, below.**

2. As related above, please specify if there any proteins binding to Mas in response to A1-7 in AT1KO ECs but not in WT ECs. If exists, please discuss about the functional relevance of the protein complex in the difference of signaling pathway between AT1KO and WT ECs.

**Thank you for suggesting this addition to the manuscript. We feel our ability to draw conclusions about AT1R independent signaling of Ang-(1-7) via Mas1 is limited by the fact that the phenotypes studied here are in fact AT1R dependent. That said, this data may be useful for generating hypotheses to test regarding AT1R independent Ang-(1-7)/Mas1 signaling. In the context of future work studying other phenotypes in the SS-AT1KO rat or SS-AT1KO cells, revisiting these data in that way may identify targets of interest; as such, full lists of the identified proteins along with scan counts and other statistics are available in the supplementary materials (S4 Table, S5 Table).**

**We have also added a supplemental table (S3 Table) that identifies proteins upregulated after Ang-(1-7) in SS-AT1KO ECs and added the following text to the manuscript:**

**Results section, page 23, lines 527-537:**

This data may also be useful in developing hypotheses regarding potential AT1R independent actions of Ang-(1-7) via Mas1. **S3 Table** shows proteins that may be related to AT1R independent signaling downstream of the Ang-(1-7) complex. This table was formed by applying the following criteria: FDR < 5%, statistically significant difference between Ang-(1-7) treated and untreated experimental groups in the SS-AT1KO endothelial cell comparison (normalized p-value < .05), greater than or equal to 6 scans detected in the Ang-(1-7) treated groups, and at least a 2-fold enrichment in the Ang-(1-7) treated group. Of particular interest may be PAK4 (log2ratio = 4.09; p = 3.31x10^-5^), which promotes cell survival [17,18], affects cell adhesion [19] and is implicated in the epithelial-mesenchymal transition in cancer [20], and PA2G4 (log2ratio = 2.58; p = 0.046), a DNA binding protein [21] which is implicated in both development [22] and a variety of cancers [23,24].

**Discussion section, page 27, lines 630-641:**

These data may be useful in developing hypotheses regarding AT1R independent Mas1 signaling. Given that the remainder of the data presented here examines only AT1R dependent effects of Ang-(1-7)/Mas1 signaling, the conclusions that can be drawn are limited. With this limitation in mind, several proteins of interest were identified as potential AT1R independent mediators of Ang-(1-7)/Mas1 signaling (**S3 Table**). Mas1 was first described as an oncogene [25] and continues to be of interest in cancer [26,27]. Therefore, PAX4 and PA2G4, both of which are implicated in the development of cancer [20,23,24] and were identified here as a potential mediators of AT1R independent Ang-(1-7)/Mas1 signaling, represent ideal candidates for further study. In particular, PAK4, which regulates cell adhesion [19] and apoptosis [17], is of interest in this context given the known effects on cell growth and/or metastasis by Ang-(1-7)/Mas1 in nasopharyngeal carcinoma [28], prostate cancer [29,30], breast cancer [31], and other cancers [32].

3. It was reported that angiotensin 1-7 directly binds to AT1 (Hypertension. 2016 Dec;68(6):1365-1374.) It is necessary to discuss about the phenotype in AT1KO rats with respect to the direct effect of angiotensin 1-7 to AT1.

**This is a thoughtful and reasonable concern noted by both reviewers. As noted in our response to Reviewer 1, we have added the following to the discussion section, page 26-27, lines 608-629:**

An important limitation of this study is the assumption that Ang-(1-7) does not exert its proangiogenic effects via binding to AT1R. This assumption is based upon our previous finding that the proangiogenic effects of Ang-(1-7) are blocked by the Mas1 antagonist A779 but not by the AT1R antagonist losartan [4]. It is possible that yet to be understood complexity will invalidate this assumption. For instance, an altered conformation of heterodimerized AT1R could allow Ang-(1-7) to bind AT1R despite the presence of losartan. However, note that the convergent, proangiogenic effects of AngII in our model were attenuated in the presence of losartan [4]. Unless AngII and Ang-(1-7) act via separate sets of AT1R, binding of AT1R by Ang-(1-7) in the presence of losartan is an unlikely mechanism for these losartan-independent proangiogenic effects. Given that the strongest evidence for Ang-(1-7) binding AT1R is displacement of AngII [6,7], we do not believe that binding of Ang-(1-7) to a separate set of AT1R that are unaffected by losartan is responsible for the proangiogenic effects observed in our model. Note that we do not discount AT1R binding as a mechanism for other effects mediated by Ang-(1-7). In fact, this possibility is of ongoing interest in the field. For instance, there is evidence that Ang-(1-7) can act as a biased ligand in cells expressing AT1R but not Mas1 [8]. There is also recent work suggesting that Ang-(1-7) does not interact directly with Mas1 [9]. This is in conflict with work demonstrating that Ang-(1-7) exerts effects via Mas1 [10], including in cells expressing Mas1 but not AT1R [11] and work that demonstrates Ang-(1-7) induced Mas1 internalization [12]. Given the ever-growing complexity of the renin-angiotensin system [13,14], including transactivation of Mas1 downstream of AngII [15], it seems unlikely that the effects of Ang-(1-7) are mediated via a single receptor. It will be important to reassess this work and other work in the field as our understanding of this complexity grows.
